# Reduction of Major Adverse Cardiovascular Events (MACE) after Bariatric Surgery in Patients with Obesity and Cardiovascular Diseases: A Systematic Review and Meta-Analysis

**DOI:** 10.3390/nu13103568

**Published:** 2021-10-12

**Authors:** Andryanto Sutanto, Citrawati Dyah Kencono Wungu, Hendri Susilo, Henry Sutanto

**Affiliations:** 1 Department of General Surgery, Faculty of Medicine, Universitas Airlangga, Surabaya 60131, Indonesia; andryanto.sutanto-2019@fk.unair.ac.id; 2 Department of General Surgery, Dr. Soetomo General Hospital, Surabaya 60286, Indonesia; 3 Department of Physiology and Medical Biochemistry, Faculty of Medicine, Universitas Airlangga, Surabaya 60131, Indonesia; 4 Institute of Tropical Disease, Universitas Airlangga, Surabaya 60115, Indonesia; 5 Department of Cardiology and Vascular Medicine, Universitas Airlangga Hospital, Surabaya 60115, Indonesia; hendrisusilo@staf.unair.ac.id; 6 Department of Physiology and Pharmacology, State University of New York (SUNY) Downstate Health Sciences University, Brooklyn, NY 11203, USA

**Keywords:** bariatric surgery, cardiovascular disease, obesity, major adverse cardiovascular events, meta-analysis, systematic review, risk factor, weight intervention

## Abstract

Cardiovascular diseases (CVDs) are the leading cause of death worldwide and obesity is a major risk factor that increases the morbidity and mortality of CVDs. Lifestyle modifications (e.g., diet control, physical exercise and behavioral changes) have been the first-line managements of obesity for decades. Nonetheless, when such interventions fail, pharmacotherapies and bariatric surgery are considered. Interestingly, a sudden weight loss (e.g., due to bariatric surgery) could also increase mortality. Thus, it remains unclear whether the bariatric surgery-associated weight reduction in patients with obesity and CVDs is beneficial for the reduction of Major Adverse Cardiovascular Events (MACE). Here, we performed a systematic literature search and meta-analysis of published studies comparing MACE in patients with obesity and CVDs who underwent bariatric surgery with control patients (no surgery). Eleven studies, with a total of 1,772,305 patients, which consisted of 74,042 patients who underwent any form of bariatric surgery and 1,698,263 patients with no surgery, were included in the systematic review. Next, the studies’ data, including odds ratio (OR) and adjusted hazard ratio (aHR), were pooled and analyzed in a meta-analysis using a random effect model. The meta-analysis of ten studies showed that the bariatric surgery group had significantly lower odds of MACE as compared to no surgery (OR = 0.49; 95% CI 0.40–0.60; *p* < 0.00001; *I*^2^ = 93%) and the adjustment to confounding variables in nine studies revealed consistent results (aHR = 0.57; 95% CI 0.49–0.66; *p* < 0.00001; *I*^2^ = 73%), suggesting the benefit of bariatric surgery in reducing the occurrence of MACE in patients with obesity and CVDs (PROSPERO ID: CRD42021274343).

## 1. Introduction

The global prevalence of cardiovascular diseases (CVDs) had doubled from 271 million cases in 1990 to 523 million cases in 2019, with mortality reaching 18.6 million cases worldwide [1], and these numbers are projected to increase in the next few years. Among all CVDs, ischemic heart disease and cerebrovascular diseases (e.g., stroke) are the major contributors to the high CVD burden. There have been a 120–137% increase in ischemic heart disease-related mortality and a 107–124% increase in stroke-related mortality in the past two decades [2]. Such a marked increase in CVD incidence is believed to be precipitated by several factors, including population aging, urbanization and technological advancement, which lead to sedentary lifestyle and obesity, two of four canonical risk factors of CVDs, together with tobacco smoking and unhealthy diet [1,2].

Obesity (a body mass index (BMI; in kg/m^2^) of more than 30 or 28 in Asian population) is known to modulate the risk for developing ischemic heart disease, cardiac arrhythmias and heart failure (HF) through several mechanisms. The excess of adipose tissue leads to insulin resistance, inflammation, activation of the renin–angiotensin–aldosterone system (RAAS) and progressive structural and electromechanical remodeling of the heart [3,4]. Therefore, reducing the accumulation of adipose tissue is essential to lower the CVD risk and burden. Lifestyle modifications (e.g., diet control, physical exercise and behavioral changes) are the first-line managements of obesity [5]. However, when these interventions fail to significantly lower the BMI, pharmacotherapies and bariatric surgery are considered [6].

**Figure 1:**
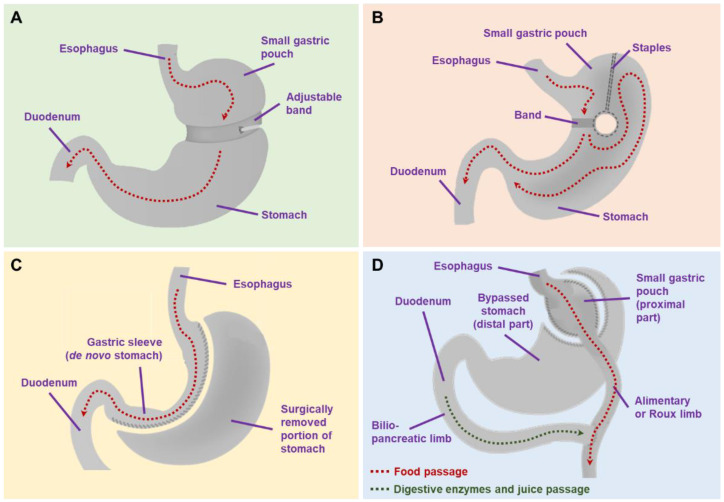
Commonly performed bariatric surgery procedures: (**A**) gastric banding; (**B**) vertical banded gastroplasty; (**C**) sleeve gastrectomy; (**D**) Roux-en-Y gastric bypass.

Bariatric surgery is by definition a surgical procedure to promote weight loss. The approach is performed by restricting gastric size to reduce the amount of food ingested and/or to facilitate malabsorption of nutrients. There are several common procedures in bariatric surgery, such as gastric banding, vertical banded gastroplasty, sleeve gastrectomy, Roux-en-Y gastric bypass and biliopancreatic diversion with/without duodenal switching [6,7]. Gastric banding (Figure 1A) is generally performed by placing a band around the stomach to restrict gastric size, whereas in the vertical banded gastroplasty (Figure 1B), the stomach is partitioned and a prosthetic is placed around the partitioned stomach. Meanwhile, a sleeve gastrectomy is done by resecting a part of the gastric body, creating a gastric sleeve that restricts the gastric size and promotes malabsorption (Figure 1C). In Roux-en-Y gastric bypass, the stomach is partitioned into proximal and distal parts. The proximal part acts as an alimentary tract and is anastomosed with the jejunum (i.e., gastrojejunostomy), while the distal part acts as a biliopancreatic limb, which is also anastomosed with the jejunum in an either end-to-side or side-to-side fashion (Figure 1D). Finally, a biliopancreatic diversion approach works quite similarly to Roux-en-Y gastric bypass by dividing the stomach into alimentary and biliopancreatic limbs, although in a biliopancreatic diversion, a gastric resection was also performed. Both Roux-en-Y gastric bypass and biliopancreatic diversion exert their functions by creating malabsorption [7].

Several studies have reported the benefits of bariatric surgery in patients with obesity, including the improvements of body fat distribution and CV risk factors, such as dyslipidemia, (pre)hypertension, insulin resistance, (pre)diabetes, non-alcoholic fatty liver disease, inflammation, vascular reactivity and obstructive sleep apnea [6]. Ample evidence also showed that bariatric surgery significantly improved the remission of type 2 diabetes mellitus (T2DM), in part through weight-independent mechanisms [8,9]. Because CVDs very often arise as a secondary yet lethal consequence of underlying chronic metabolic diseases (e.g., T2DM), conceivably through an increased prevalence of notable risk factors, such as hypertension, dyslipidemia and obesity [10], surgical procedures to treat T2DM (i.e., “metabolic surgeries”) could be advantageous to reduce CV burden, particularly when hyperglycemia is insufficiently controlled by lifestyle modifications and appropriate medications.

Typically, the reduction of those CV risk factors is expected to be followed by the reduction of Major Adverse Cardiovascular Events (MACE; i.e., composites of CV death, myocardial infarction (MI), stroke, coronary revascularization or hospitalization for HF [11,12]). However, recent studies showed conflicting results, suggesting that obesity might provide better CV outcomes in specific populations (i.e., “obesity paradox”) [13,14,15,16]. “Obesity paradox” is an epidemiological phenomenon in which overweight persons or individuals with class I obesity might have better CV outcomes and/or survival, and, potentially, less MACE occurrence compared to normal or underweighted individuals [3]. In addition, it has also been noted that bariatric surgery could exacerbate the control of certain forms of CVD (e.g., dysrhythmia and venous thromboembolism), particularly in the early postoperative period [17]. In a study by Smith et al. [18], bariatric surgery had a 0.3% mortality rate within 30 days of surgery, predominantly due to sepsis (33%), cardiac causes (28%) and pulmonary embolism (17%). Additionally, the baseline functional status of the patients could also impinge on postoperative morbidity and mortality following bariatric surgery [19].

Therefore, with the “obesity paradox” and peri/postoperative risks of CVDs in place, it is still unknown whether surgical interventions to induce significant weight loss would provide better CV outcomes, especially in high-risk individuals with previous history of CVDs. Moreover, at present, the benefit of bariatric surgery in reducing MACE in patients with obesity and CVDs remains poorly understood. Therefore, in this systematic review/meta-analysis, we sought to analyze the benefit of bariatric surgery in reducing MACE in patients with obesity and CVDs.

## 2. Materials and Methods

### 2.1. Search Strategy

This review was conducted in accordance with the Preferred Reporting Items for Systematic Reviews and Meta-Analysis (PRISMA) guideline [20]. The PRISMA checklist is available in the Supplementary Materials. Literature search was carried out electronically and the relevant studies were retrieved from PubMed/MEDLINE, ScienceDirect, Cochrane Library, Wiley Online Library and Springer databases. The search was conducted using the keywords constructed on Medical Subject Headings (MeSH) and other additional terms: “Bariatric Surgery[mesh] OR Metabolic Surgery[mesh]” AND “Cardiovascular disease*[mesh] OR Obesity” AND “Major Adverse Cardiac Event* OR Major Adverse Cardiovascular Event* OR MACE” OR “Bariatric Surgery and Long-term Cardiovascular Events”. All publications from the inception to July 2021 were evaluated. The protocol of this study was registered in PROSPERO (https://www.crd.york.ac.uk/prospero/ (accessed on 8 September 2021)), with the identification number being CRD42021274343.

### 2.2. Eligibility

We included studies that focused primarily on the comparison of MACE in patients with obesity and CVDs who underwent bariatric surgery and no surgery. Studies were opted with following inclusion criteria: (1) the primary endpoint of the studies was the occurrence of MACE (defined as all-cause mortality or the first occurrence of MI, coronary artery bypass grafting or percutaneous coronary intervention, stroke, or hospitalization for HF); (2) studies comparing surgery and no-surgery groups; (3) the study population was adults with CVDs (e.g., ischemic heart disease, hypertension, HF) and obesity, with an exclusion of persons with an age less than 18 years old or more than 80 years old, pregnancy or malignancy; (4) the full-text of the articles is accessible; (5) randomized controlled trial (RCT) or cohort studies; and (6) the studies were published in English. Studies in the form of review articles and case reports/case series were excluded.

### 2.3. Data Collection and Extraction

The literature was screened and reviewed by two independent reviewers (A.S. and H.Sut.). Any discrepancies, including the lack of concordance in the study selection evaluation, were resolved by discussion with other investigators (H.Sus. and C.D.K.W.) until reaching consensus. Screening was done by assessing the relevance of the title and abstract of the studies. Any duplication of the studies was removed using Mendeley Reference Manager. From the reference literature, the following data were taken: the type of study design, study locations (state and/or country), number of patients studied, number of patients who underwent bariatric surgery and no surgery, comorbidities (e.g., ischemic heart disease, HF, atrial fibrillation (AF), hypertension, dyslipidemia and diabetes mellitus), age of patients, BMI of patients, rate of MACE occurrence and follow-up period. The risk of bias was going to be assessed using Cochrane Risk of Bias (RoB) 2 Tool [21] and Newcastle–Ottawa Scale (NOS) [22] for RCTs and observational studies, respectively. However, in the final stage of the study selection, no suitable RCT was found; therefore, Cochrane RoB2 was not used.

### 2.4. Data Synthesis

All outcome variables were summarized and pooled in a meta-analysis using the Review Manager (RevMan) 5.4.1 software (Cochrane Collaboration). Dichotomous data were presented as the odds ratio (OR) and analyzed using the Mantel–Haenszel method. Continuous data were presented as the mean difference and analyzed using the Inverse Variance method. Heterogeneity analysis was done with the *I*^2^ test, and the data were considered heterogenous if *I*^2^ > 75% and, in this setting, a random-effect model was used. If *I*^2^ < 25%, the data were considered homogenous and a fixed-effect model was used. Publication bias was assessed visually using Begg’s funnel plot. In the presence of publication bias, the trim and fill method was used for correction. Statistical significance was considered if the two-tailed *p* value < 0.05.

## 3. Results

### 3.1. Study Characteristics

**Figure 2:**
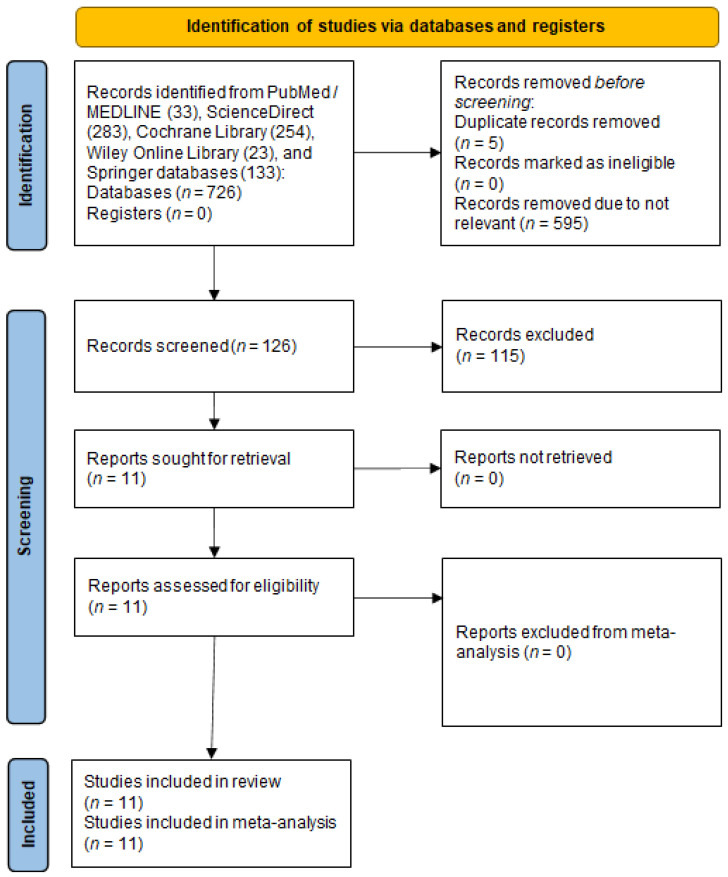
The PRISMA flow diagram of the literature search.

**Figure 3:**
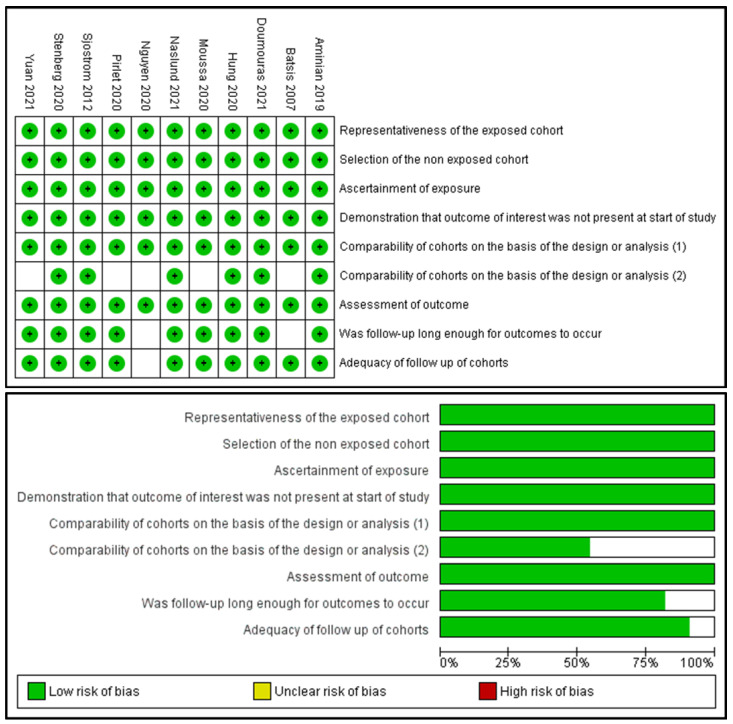
Risk of bias assessment with the Newcastle–Ottawa Scale (NOS).

A total of 726 studies were identified in the literature search, as depicted in the PRISMA flow diagram (Figure 2). Five duplicates were removed, and 595 studies were excluded because of their irrelevance to the aim of this study. One hundred and twenty-six studies were thoroughly reviewed for eligibility. After a thorough review, 115 studies were excluded; thus, 11 studies were included in the review. Of those, 10 studies were observational cohort studies and one study was a non-RCT. Subsequently, 11 studies were included in the meta-analysis (10 studies for the MACE incidence calculation and 9 studies for the confounder analysis). The risk of bias assessment is summarized in Figure 3.

From the 11 studies included in this review, there were 1,772,305 patients, consisting of 74,042 patients who underwent any form of bariatric surgery and 1,698,263 patients with no surgery. Reported bariatric procedures included Roux-en-Y gastric bypass, gastric banding, sleeve gastrectomy, biliopancreatic diversion, vertical banded gastroplasty and duodenal switch. The follow-up period of the studies ranged from 3 to 9 years. The detailed study characteristics are summarized in Table 1. Pooled means of the age of the study population were 52.55 years in the bariatric surgery group and 54.09 years in the no-surgery group. Pooled means of the BMI were 42.62 in the bariatric surgery group and 44.59 in the no-surgery group. The study population characteristics and comorbidities are listed and summarized in Table 2.

### 3.2. Highlights of the Included Studies

The study by Sjostrom et al. [23] was conducted on 2010 individuals who underwent bariatric surgery and 2037 individuals receiving conservative management. The selected patients were within the range of 37 to 60 years old and had a BMI of at least 34 for men and 38 for women. Patients with earlier surgical operation for peptic ulcer, earlier bariatric surgery, history of gastric ulcer within the last 6 months, malignancy, MI within the last 6 months, bulimic eating pattern, drug or alcohol abuse and psychiatric problems contraindicating surgery were excluded from the study. The median follow-up of the study was 14.7 years. In the study, 199 CV events (both fatal and non-fatal) in the bariatric surgery group and 234 events in the control group were reported (unadjusted HR = 0.83; 95% CI 0.69–1.00, *p* = 0.05). The incidences of fatal and non-fatal MI and stroke were also lower in the surgery group (fatal MI (HR = 0.52; 95% CI 0.31–0.89; *p* = 0.02) and total MI (HR = 0.71; 95% CI 0.54–0.94; *p* = 0.02); fatal stroke (HR = 0.34; 95% CI 0.12–1.00; *p* = 0.05) and total stroke (HR = 0.66; 95% CI 0.49–0.90; *p* = 0.008)). Interestingly, the study showed that the benefit of bariatric surgery in reducing MACE was strongly associated with a high basal plasma insulin level. In contrast, baseline BMI was not shown to be related to CV outcome of the surgical treatment benefit. 

In the study conducted by Aminian et al. [24], a total of 2287 and 11,435 individuals were included in the surgery group and in the control group, respectively. The study had a median follow-up of 3.9 years. The patients’ selection was based on inclusion criteria of age between 18 to 80 years, BMI of 30 or greater and a diagnosis of diabetes (either glycated hemoglobin (HbA1c) level of ≥6.5% or the consumption of diabetes medications). Patients with a history of solid organ transplant, severe HF (ejection fraction < 20%), malignancy or peptic ulcer were excluded. Primary endpoints of the study (i.e., the composite of first occurrence of all-cause mortality, coronary artery events, cerebrovascular events, HF, nephropathy and AF) were documented in 385 patients within the bariatric surgery group and 3243 patients in the control group, with a cumulative incidence of primary endpoint at 8-year follow-up of 30.8% in the surgery group and 47.7% in the control group (adjusted HR = 0.61; 95% CI 0.55–0.69; *p* < 0.001). Further analysis also showed that individuals in the surgery group had lower all-cause mortality (HR = 0.59; 95% CI 0.48–0.72, *p* < 0.001), HF (HR = 0.38; 95% CI 0.30–0.49; *p* < 0.001), coronary artery disease (HR = 0.69; 95% CI 0.54–0.87; *p* = 0.002), cerebrovascular disease (HR = 0.67; 95% CI 0.48–0.94; *p* = 0.02), nephropathy (HR = 0.40; 95% CI 0.31–0.52; *p* < 0.001) and AF (HR = 0.78; 95% CI 0.62–0.97; *p* = 0.03) than the control group. The bariatric surgery group was also associated with better weight and HbA1c reductions.

Stenberg et al. [25] conducted a matched cohort study involving 11,863 participants in the surgery group and 26,199 participants in the control group. The study was performed on individuals with age > 18 years old, obesity and a history of hypertension. The incidences of MACE (the first occurrence of acute coronary syndrome, cerebrovascular event, fatal CV event or unattended sudden cardiac death) were reported in 379 participants in the surgery group and 1125 participants in the control group (unadjusted HR = 0.73; 95% CI 0.65–0.82; *p* < 0.001). Individual analysis of MACE showed that the surgery group had a significantly reduced risk of acute coronary syndrome (adjusted HR = 0.53; 95% CI 0.42–0.67; *p* < 0.001) with no significance in cerebrovascular events (adjusted HR = 0.81; 95% CI 0.66–1.01; *p* = 0.063). Improvements in hypertension, diabetes and dyslipidemia were also documented in this study.

The study conducted by Pirlet et al. [26] included 116 individuals in both surgery and control groups. The study was conducted on individuals who were obese and had stable coronary artery disease. The median follow-up of the study was 8.9 years. The study showed that in the surgery group, the incidence of all-cause mortality and CV events were significantly lower than in the control group (HR = 0.55; 95% CI 0.30–0.98; *p* = 0.044 and HR = 0.64; 95% CI 0.41–0.99; *p* = 0.046, respectively). The bariatric surgery group also had a better weight reduction than the control (−28.1 ± 20.5 vs.−3.7 ± 14.4; *p* < 0.00001). Next, the study by Moussa et al. [27] opted 7402 participants, equally divided between surgery and control groups. The study included individuals with BMI > 35 without any history of previous MACE. The study showed that the bariatric surgery group had significantly lower fatal and non-fatal cardiac events (HR = 0.41; 95% CI 0.274–0.615; *p* < 0.001). Moreover, bariatric surgery was also correlated with a better weight reduction, a lower incidence of MI (HR = 0.412; 95% CI 0.280–0.606; *p* < 0.001), a reduction in incident HF (HR = 0.403; 95% CI 0.181–0.897; *p* = 0.026) and a higher diabetes resolution (HR = 3.97; 95% CI 3.20–4.93; *p*< 0.001). Naslund et al. [30] also conducted a study on patients with obesity and a previous history of MI. In this study of 566 patients, the bariatric surgery group had lower MACE (HR = 0.44; 95% CI 0.32–0.61), MI (HR 0.24; 95% CI 0.14–0.41) and new onset HF during follow-up.

Next, Hung et al. [28] conducted a study on 2872 individuals (1436 individuals in each group) of 18–55 years old who had attempted conservative methods, had a BMI > 35 with comorbidities or >40 and had no psychiatric disorders (e.g., major depression, anxiety or bulimia nervosa). The primary endpoint was hospitalization due to MI, intracranial hemorrhage, ischemic stroke or transient ischemic attack. As a result, the bariatric surgery group had significantly lower total CV events (HR = 0.168; 95% CI 0.085–0.328; *p* < 0.001), risk of MI (HR = 0.186; 95% CI 0.054–0.643; *p* = 0.008) and cerebrovascular events (HR = 0.162; 95% CI 0.073–0.360; *p* < 0.001).

In the study by Doumouras et al. [29], a total of 2638 participants were included, with 1319 individuals in each group. The study was conducted on individuals with obesity (BMI > 35) and a history of any CVD (e.g., ischemic heart disease or HF) with exclusions of age ≥ 70 years, malignancy, active substance use, pregnancy, previous solid organ (lung, liver or heart) transplant and severe liver disease with ascites. After a median follow-up of 4.9 years, the surgery group was shown to have a lower MACE occurrence (HR = 0.58; 95% CI 0.48–0.71; *p* < 0.001) and incidence of MI (HR = 0.63; 95% CI 0.42–0.96; *p* = 0.03) than the control group. Next, Batsis et al. [31] conducted a study on 197 individuals with obesity (BMI > 35) and a history of Roux-en-Y gastric bypass, and 163 individuals without surgery. The study reported 15 cases of MACE, 6 cases in the surgery group and 9 cases in the control group, although statistical significance was not reached. 

Nguyen et al. [32] analyzed the data of 1700,943 individuals from the 2012–2016 United States National Inpatient Sample (NIS) of the Healthcare Cost and Utilization Project (HCUP). A total of 1650,647 participants were included in the control group and 50,296 participants in the surgery group. They showed that the surgery group had lower MACE (6.71% vs. 13.86%; *p* < 0.001), MI (1.31% vs. 2.82%; *p* < 0.001), ischemic stroke (0.33% vs. 0.44%; *p* < 0.001) and HF (0.84% vs. 1.78%; *p* < 0.001). 

Finally, the study by Yuan et al. [33] included 308 patients with obesity (BMI > 35) who underwent Roux-en-Y gastric bypass and 701 individuals with no surgery. The study concluded that Roux-en-Y gastric bypass surgery yielded lower MACE (adjusted HR = 0.62; 95% CI 0.44–0.88; *p* = 0.008) and mortality (adjusted HR = 0.51; 95% CI, 0.26–0.96; *p* = 0.04) than the control. Moreover, the metabolic profiles (i.e., hypertension, diabetes and dyslipidemia) of the participants were also improved by bariatric surgery.

### 3.3. The Incidence of MACE

**Figure 4:**
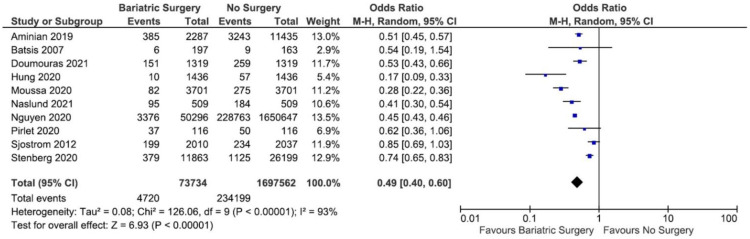
Forest plot of the MACE incidence comparing bariatric surgery with no surgery.

Out of the 11 studies included in the review, one study by Yuan et al. [33] was omitted from the first round of the meta-analysis because the OR of MACE incidence could not be calculated. From the remaining 10 studies, the incidence of MACE was pooled in a meta-analysis, and a total of 4720 cases and 234,199 cases of MACE were documented in bariatric surgery group and in the no-surgery group, respectively. The heterogeneity test revealed that the studies were heterogenous (*I*^2^ = 93%); therefore, a random-effect model was used in the meta-analysis. As a result, the meta-analysis showed that there was a significant reduction in MACE in the bariatric surgery group as compared to the no-surgery group (OR = 0.49; 95% CI 0.40–0.60; *p* < 0.00001; *I*^2^ = 93%). The meta-analysis is presented as a forest plot and displayed in Figure 4.

### 3.4. Publication Bias

**Figure 5:**
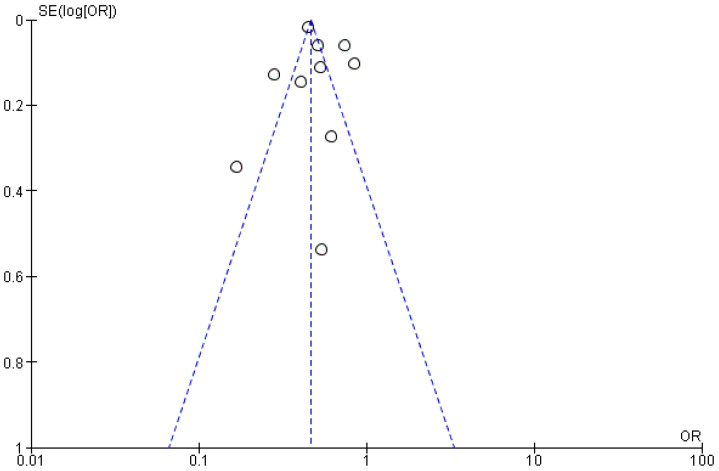
Begg’s funnel plot for publication bias assessment.

The publication bias was assessed visually using Begg’s funnel plot. As depicted in Figure 5, there was no asymmetry in the funnel plot, indicating the absence of apparent publication bias in the study.

### 3.5. Sensitivity Analysis

**Figure 6:**
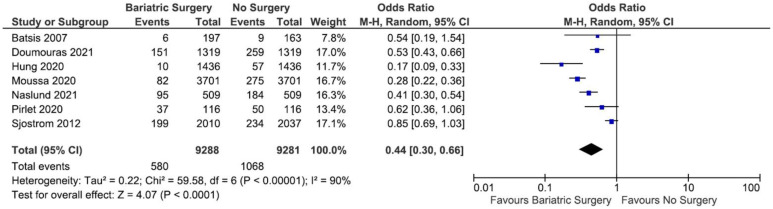
Forest plot of the MACE incidence comparing bariatric surgery with no surgery excluding studies with an unequal sample size between the arms.

Next, we carried out a sensitivity analysis by excluding studies containing an unequal sample size between the arms from the analysis. As shown in the forest plot (Figure 6), following the exclusion of studies with an unequal sample size [24,25,32], the results remained stable. In addition, when each study was sequentially excluded to assess the stability of the results, no study affected the pooled estimates.

### 3.6. Confounder Analysis

**Figure 7:**
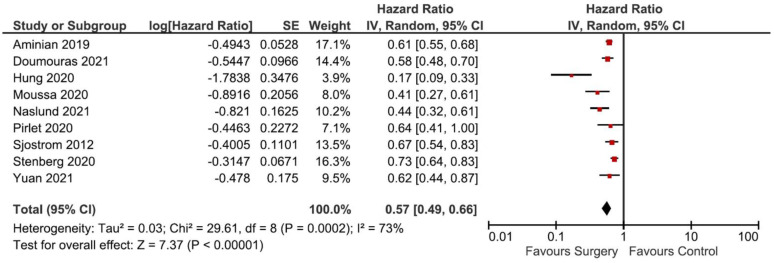
Adjusted forest plot of the MACE incidence comparing bariatric surgery with no surgery.

**Figure 8:**
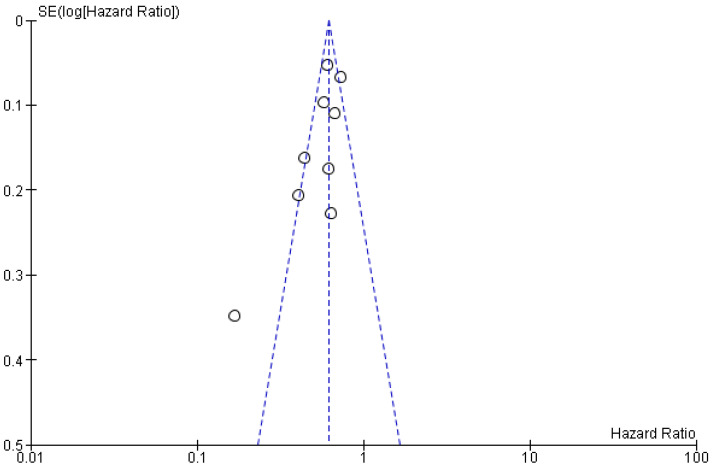
Adjusted Begg’s funnel plot for publication bias assessment.

The effect of bariatric surgery on the incidence of MACE could be influenced by other factors, including age, sex, BMI, baseline smoking behavior, diet, physical activities, comorbidities and medications. Therefore, to assess the robustness of our findings after adjusting to those confounding variables, we performed additional analyses using the data of the adjusted hazard ratio (aHR) reported in each study. Table 3 summarizes the matching characteristics, adjusted criteria for confounder exclusion and the aHR of the included studies. Of the 11 pre-included studies, we excluded 2 studies from this analysis [31,32] because of the unavailability of aHR values. Figure 7 depicts the adjusted forest plot of the MACE incidence and Figure 8 displays the adjusted Begg’s funnel plot for publication bias analysis.

## 4. Discussion

Obesity is a notable risk factor for CV and metabolic diseases, and it has been shown to increase the risk for coronary artery disease, HF, cardiac arrhythmias and diabetes mellitus [3,34]. Despite the established association between obesity and high CV risk, in recent years, several studies reported the presence of the “obesity paradox” [13,14,15,16], a phenomenon in which obesity lowers the MACE and shows a better prognosis as compared to underweighted or normal-weighted individuals. At present, this notion is still unclear and debatable [35], and the (patho)physiology behind the “obesity paradox” has not been fully elucidated. This paradox leads to a question whether a significant weight reduction in a population with obesity is advantageous to lower the incidence of MACE. The objective of our study was to evaluate the efficacy of bariatric surgery on the reduction of MACE in patients with obesity and CVDs, and our meta-analysis showed that individuals with obesity who underwent bariatric surgery had less MACE occurrence as compared to people with obesity who had no surgery, even after adjusting for confounding variables (OR = 0.49; 95% CI 0.40–0.60; *p* < 0.00001; *I*^2^ = 93% and aHR = 0.57; 95% CI 0.49–0.66; *p* < 0.00001; *I*^2^ = 73%). Importantly, this finding was consistently observed across studies, strongly indicating that bariatric surgery, and presumably a significant weight reduction in general, improved the overall CV outcomes in such specific population. 

Mechanistically, there are several mechanisms through which excess adiposity could alter the body homeostasis (e.g., cellular metabolism and CV physiology). Excess adiposity induces the expression of protein tyrosine phosphatases (PTP), such as PTP-1B and leukocyte antigen-related phosphatase (LAR), which were shown to dephosphorylate the insulin receptor and insulin receptor substrate-1 (IRS-1) in vitro, which resulted in the derangement of insulin sensitivity and energy homeostasis [36]. Adipose tissue is also an endogenous source of several proinflammatory cytokines, such as tumor necrosis factor (TNF)-α, interleukin (IL)-1, IL-6, plasminogen activator inhibitor-1 (PAI-1), C-reactive protein (CRP) and monocyte chemoattractant protein-1 (MCP-1). More recently, the possible involvement of NOD-, LRR- and pyrin domain-containing protein-3 (NLRP3) inflammasome in obesity has also been articulated [37]. Additionally, excess adiposity induces a high leptin level and consequently activates nicotinamide adenine dinucleotide phosphate (NADPH) oxidases (NOX) and induces oxidative stress [38]. Such a proinflammatory nature of adipose tissue leads to higher risks of inflammation, thrombosis and insulin resistance [36]. Excess adiposity may also activate RAAS, promoting salt and water retention and vasoconstriction. These mechanisms together with the obesity-induced autonomic nervous system remodeling could facilitate hypertension, cardiac arrhythmias (e.g., AF) and structural remodeling of the heart (i.e., HF) [3,39]. Additionally, excess adiposity could stimulate myocardial fat deposition [3]. Subsequently, obesity-induced insulin resistance may lead to T2DM, another notable risk factor for CVDs through hyperglycemia, atherosclerotic plaque formation and diabetes-induced vasculopathy [40]. 

Weight reduction, either through a surgical or non-surgical approach, has been shown to have positive effects on CV physiology. A study by Haase and colleagues [41] reported that a 13% reduction of body weight in patients with obesity significantly reduced CVD risks, including diabetes, hypertension and dyslipidemia. Moreover, studies also showed that patients with obesity who lost their weight had lower systolic blood pressure, HbA1c, low-density lipoprotein (LDL), triglycerides and CRP, and a higher high-density lipoprotein (HDL) [41,42]. Non-surgical approaches, such as lifestyle/diet modifications and pharmacological intervention, are the primary managements of obesity. However, studies reported that such non-surgical approaches had limited effectivity. For example, lifestyle modifications only yielded around a 10% weight loss in one year. Additionally, only 5.3% of the participants could maintain the attained weight loss within 8 years of observation, and the remainders regained their weight [6,43]. Therefore, a surgical approach (i.e., bariatric surgery) is highly considered in particular situations in which the non-surgical approaches fail to reach the weight loss target.

Metabolic or bariatric surgery encompasses any means of surgical approaches to induce weight loss. It is indicated in patients with a BMI > 40 or BMI > 35 with comorbidities (e.g., CVDs and diabetes mellitus) [44]. There are two main procedures in bariatric surgery: restrictive (e.g., vertical banded gastroplasty, gastric banding and sleeve gastrectomy) and malabsorptive procedures (e.g., jejunoileal bypass, duodenal switch and Roux-en-Y gastric bypass). Some procedures (e.g., jejunoileal bypass, vertical banded gastroplasty and gastric banding) are less frequent due to undesirable adverse effects, high rates of complications or reoperation, and low efficacy in the long term [44,45]. Bariatric surgery has been reported to improve CV outcomes via the improvements of CV risk factors (e.g., hypertension, diabetes and dyslipidemia) and cardiac function. Several studies have reported the benefits of bariatric surgery in glucose and fat metabolism. For example, bariatric surgery improved diabetes mellitus through the improvement of insulin sensitivity. Bariatric surgery (i.e., Roux-en-Y gastric bypass) increased the release of postprandial glucagon-like peptide-1 (GLP-1), thus increasing insulin secretion. Additionally, the sudden negative-calorie balance post-surgery induced a normalization of blood glucose within days after surgery [45]. Another study also demonstrated that bariatric surgery increased HDL cholesterol and lowered both LDL cholesterol and triglycerides [46], although the exact mechanism remains unknown. Several improvements in cardiac function were also observed in patients with obesity after bariatric surgery, including a reduction in the left ventricular mass and an improvement in the left ventricular ejection fraction [47].

Overall, despite the existence of the obesity paradox, which this study did not resolve, weight loss could merely be one of the mechanisms through which bariatric surgery affects CV outcomes [48]. There are also likelihoods of indirect positive effects of bariatric surgery on MACE. Dietary habits of postoperative patients that are influenced by psychological factors can affect weight loss and, subsequently, the risk of MACE [49]. Additionally, decreased activity of the sympathetic nervous system may modify the risk of MACE post bariatric surgery [29]. Moreover, studies exemplified that following bariatric surgery in people with obesity, the concentration of natriuretic peptides was increased and the RAAS function was normalized [50,51,52]. Finally, higher adiponectin and insulin sensitivity, as well as lower leptin, CRP and IL-6 were reported in patients post bariatric surgery [53,54]. On the whole, these effects result in the betterment of overall metabolic and CV functions, and subsequently reducing the occurrence of MACE, as observed in our meta-analysis.

Nonetheless, there are several complications of bariatric surgery, depending on the type of procedures. In general, risks of gastrointestinal obstructions (e.g., stenosis of anastomosis, intussusception and internal hernia) cannot be omitted. Specifically, in gastric banding, there is a risk for gastric necrosis. Additionally, dumping syndrome (i.e., combinations of sweating, dizziness, palpitations, abdominal pain, nausea, vomiting and/or diarrhea due to rapid gastric emptying) could also occur following bariatric surgery [55].

At last, there are several limitations of this systematic review/meta-analysis. First, our study did not differentiate outcomes based on different bariatric procedures because of the insufficiency of available data. Second, the potential interactions among covariates were not extensively explored due to data inaccessibility. Third, the unavailability of RCT also limits the interpretability of our findings. In the future, RCTs would be needed to support/confirm our findings. Additionally, further analysis based on individual type of bariatric procedures and a more detailed analysis on individual MACE components are warranted.

## 5. Conclusions

Our systematic review/meta-analysis highlighted a significantly lower MACE in patients with obesity and CVDs who underwent bariatric surgery as compared to patients with no surgery. Such a MACE-lowering effect could be due to a reduction in CV risk/burden, through the normalization of glucose and fat metabolism, the improvement of cardiac function and the improvement of overall CV outcomes.

## Figures and Tables

**Table 1:** Characteristics of the included studies.

Study	Design	Study Location	Sample Size	Surgical Procedure (%)	Follow-Up Period (Mean ± SD/Median (IQR), y)	Population	REF
Sjostrom et al., 2012	Non-RCT	Sweden	2010 surgery	Gastric Bypass: 265 (13.2) Gastric Banding: 376 (18.7) Vertical Banded Gastroplasty: 1360 (68.1)	14.7	Patients aged 37 to 60 years and with BMI of at least 34 for men and at least 38 for women.	[23]
			2037 control	-	14.7		
Aminian et al., 2019	Matched Cohort Study	Florida, Ohio, USA	2287 surgery	Roux-en-Y Gastric Bypass: 1443 (63) Sleeve Gastrectomy: 730 (32) Gastric Banding: 109 (5) Duodenal Switch: 5 (0.2)	3.3 (1.2–6.3)	Patients with age 18–80 years, BMI ≥ 30, and diabetes.	[24]
			11,435 control		4.0 (2.1–6.1)		
Stenberg et al., 2020	Matched Cohort Study	Sweden	11,863 surgery	Gastric Bypass: 10,692 (90.1) Sleeve Gastrectomy: 1171 (9.9)	5.09 ± 2.53	Patients with morbid obesity and hypertension	[25]
			26,199 control	-	5.06 ± 2.55		
Pirlet et al., 2020	Matched Cohort Study	Quebec, Canada	116 surgery	Gastric Bypass: 3 (2.6) Biliopancreatic diversion with duodenal switch: 44 (38) Sleeve gastrectomy: 67 (58) Duodenal Switch only: 2 (1.7)	8.9 (6.3–14.2)	Patients with history of coronary artery disease (CAD) and obesity	[26]
			116 control	-	8.9 (6.3–14.2)		
Moussa et al., 2020	Matched Cohort Study	UK	3701 surgery	N/A	11.2	Patients with BMI ≥ 35 with exclusion of previous MACE	[27]
			3701 control	-	11.2		
Hung et al., 2020	Matched Cohort Study	Taiwan	1436 surgery	N/A	7.5	Patients with BMI > 35 with co-morbidities or >40	[28]
			1436 control	-	7.5		
Doumouras et al., 2021	Matched Cohort Study	Ontario, Canada	1319 surgery	Gastric Bypass: 1049 (79.5) Sleeve Gastrectomy: 270 (20.5)	4.65 (3.09–6.28)	Patients with BMI > 35 with a comorbidity or BMI ≥ 40	[29]
			1319 control	-	4.38 (2.83–6.09)		
Naslund et al., 2021	Matched Cohort Study	Sweden	509 surgery	Roux-en-Y Gastric Bypass: 465 (91) Sleeve Gastrectomy: 44 (9)	4.6 (2.7–7.1)	Patients with severe obesity and history of MI	[30]
			509 control	-	4.6 (2.7–7.1)		
Batsis et al., 2007	Retrospective Cohort	Olmsted, Minnesota, USA	197 surgery	Roux-en-Y Gastric Bypass: 197 (100)	3.3 ± 2.6	Patients with BMI > 35	[31]
			163 control		3.3 ± 2.6		
Nguyen et al., 2020	Retrospective Cohort	USA	50,296 surgery	N/A	N/A	Adult patients with class II (BMI 35.0 to 39.9) or class III obesity (BMI > 40)	[32]
			1650,647 control	-	N/A		
Yuan et al., 2021	Retrospective Cohort	Minnesota, USA	308 surgery	Roux-en-Y Gastric Bypass: 308 (100)	4.6 (2.7–7.1)	Patients with class II-III obesity (BMI > 35)	[33]
			701 control	-	4.6 (2.7–7.1)		

**Table 2:** Characteristics of the study population.

Study	Group	Age (Mean ± SD/Median (IQR), y)	BMI (Mean ± SD/Median (IQR), y)	Ischemic Heart Disease (%)	HF (%)	AF (%)	Hypertension (%)	Dyslipidemia (%)	Diabetes Mellitus (%)
Sjostrom et al., 2012 [23]	Surgery	≤47.8: 55% ˃47.8: 45%	≤40.8: 40% ˃40.8: 60%	N/A	N/A	N/A	991 (49.3)	N/A	345 (17.2)
	Control	≤47.8: 45% ˃47.8: 55%	≤40.8: 60% ˃40.8: 40%	N/A	N/A	N/A	725 (35.6)	N/A	262 (12.8)
Aminian et al., 2019 [24]	Surgery	52.5 (43.7–60.5)	45.1 (40–51.8)	237 (10.4)	238 (10.4)	152 (6.6)	1953 (85.4)	1686 (73.7)	2287 (100)
	Control	54.8 (46.2–62.5)	42.6 (39.4–47.2)	1104 (9.7)	1342 (11.7)	701 (6.1)	8565 (74.9)	7457 (65.2)	11,435 (100)
Stenberg et al., 2020 [25]	Surgery	52.1 ± 7.46	41.9 ± 5.43	N/A	N/A	N/A	11,863 (100)	4437 (37.4)	3328 (28.1)
	Control	54.6 ± 7.12	N/A	N/A	N/A	N/A	26,199 (100)	7802 (29.8)	2690 (10.3)
Pirlet et al., 2020 [26]	Surgery	52.9 ± 7.2	42.0 ± 6.1	116 (100)	6 (5.2)	3 (2.6)	94 (81)	96 (83)	57 (49)
	Control	52.1 ± 8.4	41.2 ± 6.7	116 (100)	9 (7.8)	5 (4.3)	94 (81)	101 (87)	59 (51)
Moussa et al., 2020 [27]	Surgery	36 (29–44)	40.3 (36.6–43.9)	N/A	N/A	N/A	1928 (52.1)	50 (1.4)	922 (25)
	Control	36 (29–44)	40.5 (37.1–45.5)	N/A	N/A	N/A	1822 (49.2)	39 (1.1)	881 (23.9)
Hung et al., 2020 [28]	Surgery	32.39 ± 8.6	N/A	N/A	N/A	N/A	109 (7.59)	50 (3.48)	74 (5.15)
	Control	32.27 ± 9.3	N/A	N/A	N/A	N/A	116 (8.08)	57 (3.97)	76 (5.29)
Doumouras et al., 2021 [29]	Surgery	55.4 ± 7.43	48.0 ± 8.04	1202 (91.1)	274 (20.8)	105 (8)	1098 (83.2)	N/A	775 (58.8)
	Control	56.5 ± 7.85	46.7 ± 13.8	1201 (91.1)	274 (20.8)	80 (6.1)	1061 (80.4)	N/A	745 (56.5)
Naslund et al., 2021 [30]	Surgery	53.0 ± 7.0	40.6 ± 4.4	509 (100)	53 (10.4)	29 (5.7)	332 (65.5)	N/A	209 (41.1)
	Control	53.2 ± 7.4	39.7 ± 4.7	509 (100)	97 (19.1)	49 (9.6)	365 (71.7)	N/A	229 (45)
Batsis et al., 2007 [31]	Surgery	44.0 ± 9.9	49.5 ± 8.9	N/A	N/A	N/A	105 (53.3)	114 (57.9)	61 (31)
	Control	43.4 ± 11.2	44.0 ± 5.7	N/A	N/A	N/A	80 (49.1)	97 (59.5)	41 (25.2)
Nguyen et al., 2020 [32]	Surgery	52.9 ± 12.1	N/A	6776 (13.47)	6743 (13.41)	6552 (13.03)	26,793 (53.27)	14,886 (29.6)	17,059 (33.92)
	Control	54.1 ± 15.6	N/A	345,014 (20.9)	389,677 (23.61)	249,228 (15.1)	804,920 (48.76)	614,021 (37.2)	748,484 (45.34)
Yuan et al., 2021 [33]	Surgery	44.2 ± 10.5	46.4 ± 6.5	15 (4.9)	1 (0.3)	N/A	136 (44.2)	134 (43.5)	65 (21.1)
	Control	43.6 ± 12.6	44.8 ± 6.9	64 (9.1)	25 (3.6)	N/A	393 (56.1)	372 (53.1)	271 (38.7)

HF, heart failure; AF, atrial fibrillation.

**Table 3:** Matching characteristics and adjusted results of the included studies.

Studies	Matching Method	Adjusted Criteria (Confounder Exclusion)	Results
Sjostrom, et al., 2012 [23]	A matched control group of participants was created by an automatic matching program using 18 matching variables.	Adjusted for sex, age, history of stroke or MI, diabetes, insulin level, smoking history, BMI, waist circumference, hip circumference, systolic BP, total cholesterol, HDL cholesterol, triglycerides, lipid-lowering medication, antihypertensive medication.	aHR = 0.67; 95% CI 0.54–0.83; *p* < 0.001
Aminian, et al., 2019 [24]	Each surgical patient was matched with a propensity score by the nearest-neighbor method to 5 nonsurgical patients from a logistic regression model with a logit link function based on 7 a priori–identified potential confounders including the index date, age at index date, sex, BMI at index date (categorized as 30–34.9, 35–39.9, ≥40), location, insulin use, and presence of diabetes-related end-organ complications.	Adjusted for index date, sex, age, BMI, weight, race, annual income, smoking status, location of patients, medical history (hypertension, dyslipidemia, peripheral neuropathy, HF, coronary artery disease, chronic obstructive pulmonary disease, nephropathy, atrial fibrillation, peripheral artery disease, MI, cerebrovascular disease, ischemic stroke and dialysis).	aHR = 0.61; 95%CI 0.55–0.69; *p* < 0.001
Stenberg, et al., 2020 [25]	1:10 matched group of non-operated–on individuals, based on age, sex, and regional area of residence in Sweden.	Adjusted for duration of hypertension, comorbidities, and education.	aHR = 0.73; 95% CI 0.64–0.84; *p* < 0.001
Pirlet et al., 2020 [26]	Matched based on propensity score based on age, sex, BMI, weight, status of dyslipidemia, hypertension, diabetes, history of smoking, atrial fibrillation, HF, cancer, stroke, chronic obstructive pulmonary disease, chronic kidney disease, history of MI, history of ercutaneous coronary intervention, revascularization for MI, revascularization for unstable angina.	Adjusted from baseline characteristic not balanced in propensity score matching (History of MI and weight).	aHR = 0.64; 95% CI 0.41–0.99; *p* = 0.046
Moussa et al., 2020 [27]	Matched based on age, gender, and baseline BMI.	Adjusted for hypertension, hyperlipidaemia, diabetes, smoking, alcohol use, cocaine use, exercise and use of medications, such as beta-blockers, calcium channel blockers, angiotension converting enzyme inhibitors or angiotensin receptor blockers, statins, aspirin and hormone replacement therapy.	aHR = 0.410, 95% CI 0.274–0.615; *p* < 0.001
Hung et al., 2020 [28]	Matched 1:4 with patients in the surgical group by propensity-score matching, based on age and sex.	Adjusted for age, sex, Charlson Comorbidity Index (CCI), comorbidities (i.e., diabetes, hypertension, hyperlipidemia and gout).	aHR = 0.168; 95% CI 0.085–0.328; *p* < 0.001
Doumouras et al., 2021 [29]	Matched based on demographic status, history of smoking, history of diabetes mellitus, cardiac disease, stroke, hypertension, substance abuse, eating and mood disorder, liver and renal disease.	Adjusted for age, BMI, sex, immigrant status, income, rurality, diabetes status, overall cardiac history, stroke, chronic obstructive pulmonary disease, hypertension, sleep apnea, renal disease, smoking status, previous malignancy, substance abuse, self-harm, mood disorder, cancer screening (colon, breast, cervical) and health care use in previous year (family physician, hospital inpatient, emergency room visit, specialist visit).	aHR = 0.58; 95% CI 0.48–0.71; *p* < 0.001
Naslund et al., 2021 [30]	Matched 1:1 based on sex, age ( ± 3 years), year of MI ( ± 3 years), and BMI ( ± 3) to a control with MI registered in SWEDEHEART.	Adjusted for BMI, smoking, hypertension, chronic kidney disease, peripheral artery disease, HF, atrial fibrillation, chronic obstructive pulmonary disease, cancer disease within 3 years, and treatment with aspirin, P2Y12 receptor blockers, and statins.	aHR = 0.44; 95% CI 0.32–0.61
Yuan et al., 2021 [33]	Matched based on age, sex and BMI.	Adjusted for age and sex.	aHR = 0.62; 95% CI 0.44–0.88; *p* = 0.008
Batsis et al., 2007 [31]	Not matched	No adjustment	OR = 0.54; 95% CI 0.19–1.54
Nguyen et al., 2020 [32]	Not matched	Adjusted for gender, hospital region, all patients refined diagnosis related groups severity and risk of mortality, diabetes, hypertension, hyperlipidemia, chronic kidney disease, prior MI, peripheral arterial disease, chronic obstructive pulmonary disease, pulmonary arterial hypertension, atrial fibrillation and smoking.	aOR = 0.62; 95% CI 0.60–0.65; *p* < 0.001

## Data Availability

The data that support the findings of this study are available upon reasonable request.

## Supplementary Materials

The following are available online at https://www.mdpi.com/article/10.3390/nu13103568/s1, PRISMA checklist.
